# Circulating early biomarkers of atherogenesis in participants of the Longitudinal Study of Adult Health (ELSA-Brasil) without diabetes or cardiovascular disease

**DOI:** 10.1590/2359-3997000000205

**Published:** 2016-08-31

**Authors:** Bianca de Almeida-Pititto, Fernando Flexa Ribeiro-Filho, Sandhi Barreto, Bruce B. Duncan, Maria Inês Schmidt, Paulo A. Lotufo, Isabela M. Bensenor, Sandra R. G. Ferreira

**Affiliations:** 1 Faculdade de Saúde Pública Universidade de São Paulo São Paulo SP Brasil Faculdade de Saúde Pública, Universidade de São Paulo (FSP-USP), São Paulo, SP, Brasil; 2 Departamento de Medicina Interna Universidade Federal do Pará Belém PA Brasil Departamento de Medicina Interna, Universidade Federal do Pará (UFPA), Belém, PA, Brasil; 3 Faculdade de Medicina Universidade Federal de Minas Gerais Belo Horizonte MG Brasil Faculdade de Medicina, Universidade Federal de Minas Gerais (UFMG), Belo Horizonte, MG, Brasil; 4 Faculdade de Medicina Universidade Federal do Rio Grande do Sul Porto Alegre RS Brasil Faculdade de Medicina, Universidade Federal do Rio Grande do Sul (UFRGS), Porto Alegre, RS, Brasil; 5 Departamento de Medicina Interna Universidade de São Paulo São Paulo SP Brasil Departamento de Medicina Interna, Universidade de São Paulo (USP), São Paulo, SP, Brasil

**Keywords:** Biomarkers, atherogenesis, adiposity, age, sex, insulin resistance

## Abstract

**Objective:**

Our aim was to describe the distribution of selected biomarkers according to age and sex, adjusted for HOMA-IR and adiposity, in a subset of middle-aged individuals of Brazilian Longitudinal Study of Adult Health-ELSA without diabetes mellitus or CVD.

**Subjects and methods:**

This cross-sectional study was conducted in 998 participants of the ELSA-Brasil without diabetes and/or cardiovascular disease. In addition to the traditional risk factors, several biomarkers concentrations were compared according to sex, age groups (35-44; 45-54 yrs) and HOMA-IR tertiles. Linear regression was used to examine independent associations of sex and age with selected novel biomarkers, adjusted for body adiposity and HOMA-IR.

**Results:**

Fifty-five percent were women. Men had higher mean values of body mass index, waist circumference, blood pressure, plasma glucose, HOMA-IR, worse lipid profile and higher E-selectin and lower leptin concentrations than women; while women had higher levels of HDL-cholesterol and leptin than men. Mean values of waist circumference, systolic BP, plasma glucose and apolipoprotein B (Apo B) increased with age in both sexes. Leptin and E-selectin concentrations increased across HOMA-IR tertiles. Independent associations of Apo B with age were found only in male sex, while of leptin with body mass index and HOMA-IR, and of E-selectin with HOMA-IR in both sexes.

**Conclusions:**

In conclusion, our data indicate age, sex, adiposity and, consequently, insulin resistance, influence circulating levels of Apo B, leptin and E-selectin, suggesting that those aspects should be taken into consideration when assessing these parameters for research or clinical purposes in individuals at relatively low cardiometabolic risk.

## INTRODUCTION

Cardiovascular diseases (CVD) are major causes of death in both sexes (1). Mortalities due to CVDs are declining, but they are still the leading causes of death in several developed and developing countries (2-5). In Brazil, population aging has resulted in an increased chronic disease burden, but age-standardized mortalities from CVDs have declined (6). The decline has mostly been attributed to the control of traditional cardiovascular risk factors such as smoking, elevated blood pressure levels and lipids concentrations (7-9). However, obesity is increasing worldwide, and diabetes mellitus is considered one of the major epidemics of the century (10).

Until their fifties, women have lower rates of CVDs than men of the same age (11). This apparent protection from CVDs has been attributed to adequate levels of endogenous estrogens rather than to ageing. Several years after menopause, cardiovascular mortality rates in women show a steep increase (12). Despite disparities in the detection of CVDs, treatment and prognosis between sexes, basic differences in physiology and physiopathology definitely contribute to different cardiovascular morbidity and mortality rates in men and women. Gender differences in the impact of traditional risk factors are recognized. Female smokers are significantly more likely to suffer an ischemic heart disease than men (13). The impact of diabetes on the risk of coronary death is significantly greater for diabetic women than diabetic men (14). More recently, studies investigated whether novel biomarkers could particularly influence cardiovascular risk in women (15). Nowadays, distinct prediction models of cardiovascular events have been proposed for each sex (15-18).

Numerous early markers of atherogenesis, mainly inflammatory and hemostatic markers, have been described during the last decade. Markers of inflammation, insulin resistance and endothelium dysfunction are increased in obesity (19) and are considered underlying mechanisms of the atherosclerotic disease (20). Attempts to improve risk prediction by adding inflammatory markers, such as the C-reactive protein, to existing risk scores have been reported (15,18). Also, studies have investigated the role of other inflammatory and endothelial dysfunction markers in predicting diabetes and CVDs. However, limited data are reported concerning the distribution of these circulating biomarkers in individuals with lower cardiometabolic risk and how those values vary according to age and sex (21,22).

In Brazil, a large multicenter cohort study, the Brazilian Longitudinal Study of Adult Health (ELSA-Brasil), was conducted to evaluate risk factors associated with CVDs and diabetes mellitus in civil servants from six sites (23). The availability of traditional cardiovascular risk factors and a broad spectrum of circulating biomarkers of atherogenesis in the ELSA-Brasil baseline data represent an opportunity to describe these biomarkers in a large sample of middle-aged adults.

Our aim was to describe the distribution of selected biomarkers according to age and sex, adjusted for homeostasis model assessment (HOMA-IR) and adiposity, in a subset of middle-aged participants of ELSA-Brasil without diabetes mellitus or a CVD.

## SUBJECTS AND METHODS

This is a cross-sectional study that was developed based on baseline data from the multicenter cohort study ELSA-Brasil. A subsample of the participants of an ELSA-Brasil research center, located in the University of São Paulo, was included. The main objective of ELSA-Brasil is to investigate the incidence and progression of diabetes and CVDs and their biological, behavioral, environmental, occupational, psychological and social factors. Details on objective and methodological aspects were previously reported (23). Briefly, all active or retired employees of six Brazilian universities, aged 35-74 years, were eligible for the cohort study. The first examinations of 15,105 individuals (54% women) were carried out from August 2008 through December 2010. A random sample of 1,000 individuals of the research center from the University of São Paulo without diabetes and CVDs aged between 35 and 54 years was drawn from all 5,061 participants of ELSA-Brasil in São Paulo. Two individuals were excluded from the final sample because insufficient aliquots were frozen for the analysis of novel biomarkers. The institutional ethics committee approved the study, and written consent was obtained from all individuals.

Participants had an initial interview at the job site and were then scheduled for clinical exams and laboratory tests in the research center. Body weight and height were measured using calibrated electronic scales and a fixed rigid stadiometer, and participants wore light clothing and no shoes. Body mass index (BMI) was calculated as weight (kilograms) divided by squared height (meters). Waist circumference was measured with an inextensible tape according to the World Health Organization technique (24). Blood pressure (BP) was taken three times after a 5-minute rest in the sitting position. The mean of the second and third measurements was used in the analyses (25).

After overnight fasting, participants underwent a 2-hour, 75-gram oral glucose tolerance test. The blood samples were taken for several determinations at the University Hospital of the University of Sao Paulo (USP). For all the laboratory procedures, the ELSA-Brasil study had rigorous standards that are well described in a paper about pre-analytical aspects, including choosing the collection place, identifying and preparing participants, identifying collection site(s), applying and timing tourniquets, administering venipuncture techniques, ordering the collection of tubes, collecting by volume, handling tubes and processing biological samples and centrifuging, freezing and transporting biological material (26,27).

Plasma glucose was measured using the hexokinase method (ADVIA Chemistry; Siemens, Deerfield, Illinois, USA). Total cholesterol was assessed using the cholesterol oxidase method (enzymatic colorimetric). HDL-cholesterol was assessed using the homogeneous colorimetric method without precipitation. Triglycerides were assessed using the glycerol-phosphate peroxidase method according to Trinder (enzymatic colorimetric) assays (ADVIA Chemistry; Siemens, Deerfield, Illinois, USA). The low-density lipoprotein cholesterol concentrations were calculated with the Friedewald equation. The American Diabetes Association’s diagnostic criteria were used to diagnose categories of glucose tolerance (28). The HOMA-IR was used to assess insulin resistance (29). Aliquots were frozen at -80^o^C for further determinations of hormone apolipoproteins, markers of inflammation and endothelium adhesion molecules (30).

Insulin was determined using enzyme-linked immunoenzymatic assay (ELISA) (Siemens, Tarrytown, USA) and high-sensitivity C-reactive protein through immunochemistry (Dade Behring, Siemens, Marburg, Germany). ELISA kits were also used for the determination of adiponectin and leptin (Enzo Life Sciences, Farmingdale, NY, USA), apolipoprotein B (Apo B), E-selectin and transforming growth factor β1 (TGF-β1) (Abnova Corp, Taipei, Taiwan), lipoprotein (a) (Lp(a)) (ALPCO Diagnostics, Salem, NH, USA), asymmetric dimethylarginine (ADMA) and fibrinogen (Affinity Biologicals Inc., Hamilton, ON, Canada). Interleukin-6 (IL-6), interleukin-10 (IL-10), tumor necrosis factor α (TNF-α) and monocyte chemoattractant protein-1 (MCP-1) were determined using the Bio-Plex^®^ Pro Human Cytokine 4-plex assay panel (Biorad, São Paulo, SP, Brazil). Intra-assay coefficients of variation ranged from 1.8 to 7.2, except for those of TGF-β1, which reached 10%. Inter-assay coefficients varied from 0.9 to 9.1 except for TGF-β1 (12.7%) and E-selectin (14.4%).

### Statistical analysis

Descriptive data were either expressed as means and standard deviations (SDs) or as medians (interquartile range). Distributions of the biomarker concentrations were skewed and thus log-transformed before analysis to achieve normality. Biomarker means by sex and age group (35-44 and 45-54 years) were compared using an unpaired student t-test when normally distributed. Frequencies were compared using a chi-squared test. Participants were categorized into tertiles of HOMA-IR, and data were compared via ANOVA. A Pearson (r) or Spearman (rho) coefficient was used to test correlations between each biomarker and measures of obesity (BMI and waist circumference) according to sex. Whenever a significant correlation was found, a sex-specific multivariate analysis using multiple linear regression was used to examine independent associations of age, adiposity and HOMA-IR with the selected novel biomarkers.

Sensitivity analyses, excluding participants who used medications (antihypertensive and/or lipid reducing agents) or smoked, were conducted to avoid their influence on the associations found. Considering that conditions related to clinical inflammation could interfere in measurements of these biomarkers, some procedures were performed. For the blood sample collection, individuals who self-reported infectious diseases were invited to return to the clinic after they recovered. However, chronic inflammatory disorders were not exclusion criteria in our sample. Participants reported rheumatologic diseases (n = 13), rheumatoid arthritis (n = 12), lupus (n = 2), arthrosis (n = 55) and arthritis (n = 24). In order to control for these potential confounding factors, sensitivity analyses considering these conditions were performed and nonsteroidal anti-inflammatory drugs (n = 1), acetylsalicylic acid (n = 7), hormonal anti-inflammatory drugs (n = 1) and CRP levels > 9.9 mg/L (n = 28) were used. Less than 5% of women in the sample were using hormone replacement therapy for menopause, which had a negligible influence on results. Pre-diabetes status was identified in 661 individuals who had similar profiles to the whole group [mean age of 46.3 (SD 4.8) years; 53% of men, mean BMI was 26.9 (SD 4.2) kg/m^2^]. The results of sensitivity analyses did not differ when pre-diabetic individuals or those who used anti-hypertensive drugs (n = 120) were excluded. Therefore, results excluding these participants were not shown. All statistical analyses were performed using the Statistical Package for Social Sciences, version 17.0 for Windows (SPSS Inc., Chicago, Illinois, USA). A p-value < 0.05 was considered statistically significant.

## RESULTS

The mean age of the 998 individuals studied was 45.8 (SD 4.9) years; 55% were women and 19% had a BMI ≥ 30 kg/m^2^. Of them, 18.4% were current smokers, being 22.6% of men and 15.0% of women (p < 0.01).

Men had higher mean values of waist circumference, BP levels, fasting plasma glucose levels and HOMA-IR and worse lipid profiles than women (Table 1). Mean values of waist circumference, systolic BP, fasting plasma glucose and Apo B increased in the older group (45-54 years) of both sexes. In women alone, total cholesterol, LDL-cholesterol and triglyceride values were higher in the older group. Men had higher E-selectin while women had higher leptin levels. In women, HOMA-IR, Il-6 and TNF-α values were higher in the older group, while in men, leptin and CRP values were higher in the same age group. No other biomarker differed between the age groups of both sexes.

**Table 1:** Mean (SD) values of cardiovascular risk factors and biomarkers according to sex and age group in a subset participants of ELSA-Brasil

	Women			Men		
	
	Total	35 - 44 yrs	45 - 54 yrs	Total	35 - 44 yrs	45 - 54 yrs
BMI (kg/m^2^)	26.3 (4.2)	25.8 (4.2)	26.6 (4.2)^£^	26.6 (4.0)	26.3 (4.2)	26.8 (4.0)
Waist circumference (cm)	83.3 (10.7)	81.2 (10.1)	84.5 (10.9)^£^	91.3 (10.7)^π^	89.8 (11.1)	92.2 (10.4)^£^
Systolic BP (mmHg)	112 (13)	109 (12)	113 (13)^£^	122 (14)^π^	119 (12)	124 (15)^£^
Diastolic BP (mmHg)	72 (10)	71 (10)	73 (10)	78 (10)^π^	76 (9)	80 (11)^£^
Plasma glucose (mg/dL)	100 (7)	98 (7)	102 (8)^£^	105 (8)^π^	104 (7)	106 (9)^£^
HOMA-IR^#^	1.7 (1.2)	1.5 (1.1)	1.8 (1.3)^£^	2.1 (1.8)^π^	1.9 (1.7)	2.2 (1.8)
Total cholesterol (mg/dL)	208 (36)	197 (34)	214 (36)^£^	212 (37)	207 (34)	214 (38)
LDL-cholesterol (mg/dL)	126 (31)	118 (29)	131 (31)^£^	132 (33)^π^	129 (30)	134 (34)
HDL-cholesterol (mg/dL)	60 (13)	59 (13)	60 (13)	50 (11)^π^	48 (8)	51 (13)^£^
Triglycerides (mg/dL)	110 (61)	101 (61)	115 (61)^£^	150 (86)^π^	153 (39)	148 (82)
Apolipoprotein B (mg/dL)^#^	187 (171)	154 (132)	206 (188)^£^	205 (196)^π^	159 (114)	232 (228)^£^
Lipoprotein (a) (mg/dL)^#^	17.1 (25.8)	15.6 (11.2)	18.0 (31.5)	16.2 (14.1)	17.5 (16.8)	15.4 (12.1)
Fasting Insulin (µUI/mL)^#^	6.8 (4.9)	6.3 (4.4)	7.0 (5.2)	7.9 (6.7)	7.6 (6.5)	8.2 (6.8)
Leptin (ng/mL)^#^	25.3 (38.4)	25.7 (28.4)	25.0 (43.5)	10.5 (9.8)^π^	9.2 (8.1)	11.3 (10.6)^£^
Adiponectin (mcg/mL)^#^	13.6 (18.1)	14.6 (26.3)	13.0 (10.4)	12.1 (14.8)	11.8 (13.0)	12.3 (15.8)
C-reactive protein (mg/L)^#^	2.7 (3.5)	2.6 (3.0)	2.8 (3.8)	2.2 (4.2)^π^	1.7 (2.4)	2.5 (5.0)^£^
Interleukin-6 (pg/mL)^#^	19.3 (36.8)	14.5 (10.7)	22.2 (45.6)^£^	18.7 (41.2)	18.0 (28.0)	19.2 (47.4)
Interleukin-10 (pg/mL)^#^	10.6 (60.3)	17.2 (95.5)	6.8 (20.2)	10.5 (59.3)	9.3 (58.6)	11.1 (59.7)
TNF-α (pg/mL)^#^	24.2 (84.4)	14.9 (23.2)	29.8 (104.9)^£^	28.1 (147.2)	20.3 (52.5)	32.7 (181.4)
MCP-1 (pg/mL)^#^	42.4 (33.5)	42.1 (34.1)	42.5 (33.2)	40.6 (29.8)	41.4 (31.7)	40.3 (28.6)
TGF-β1 (pg/mL)^#^	19.6 (19.0)	17.9 (16.1)	20.6 (20.6)	22.3 (9.4)	18.3 (14.6)	24.6 (118.5)
E-selectin (ng/mL)^#^	80.9 (54.5)	79.5 (51.9)	81.8 (56.1)	93.7 (61.9)^π^	91.4 (59.7)	95.0 (63.2)
ADMA (µmol/L)^#^	0.20 (0.12)	0.21 (0.12)	0.19 (0.12)	0.20 (0.10)	0.19 (0.12)	0.20 (0.09)
Fibrinogen (g/L)	1.6 (0.4)	1.6 (0.3)	1.6 (0.4)	1.6 (0.4)	1.6 (0.3)	1.6 (0.4)

BP: blood pressure; TNF-α: tumor necrosis factor alpha; MCP-1: monocyte chemoattractant protein-1; TGF-β1: transforming growth factor β1; ADMA: asymmetric dimethylarginine. # log-transformed variables for analyses. Student t test used to compare sex and age groups. ^π^ P < 0.05 for men versus women ^£^ P < 0.05 for 35-44 versus 45-54 years.

**Figure 1:**
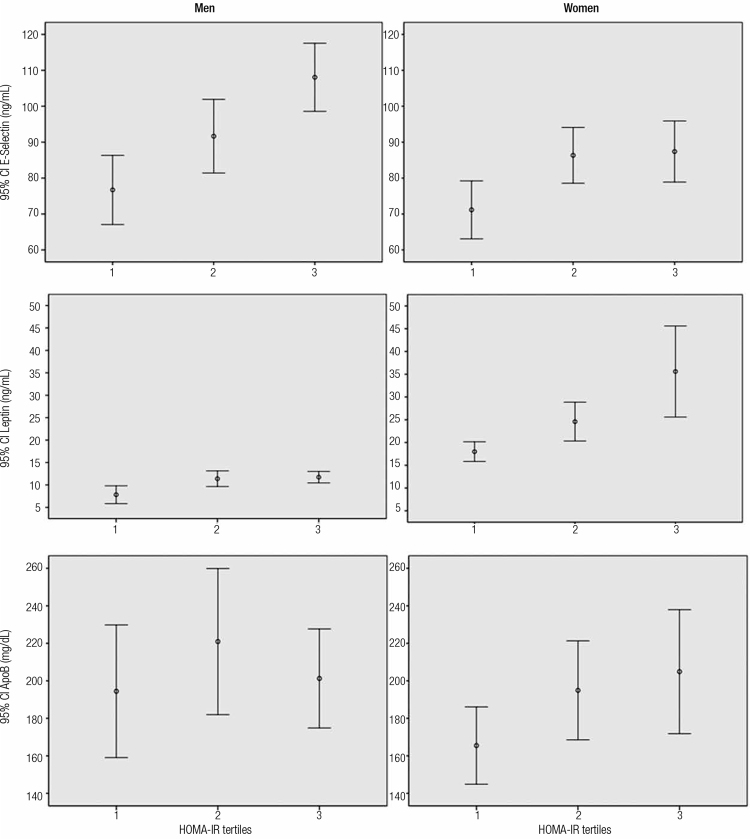
Means and 95% confidence intervals of E-selectin, leptin and apolipoprotein B across tertiles of HOMA-IR according to sex.

Biomarkers were compared across tertiles of HOMA-IR (Figure 1). E-selectin and leptin concentrations gradually increased across tertiles in men (ANOVA, p < 0.001 for E-selectin and leptin) and women (ANOVA, p = 0.007 for E-selectin and p < 0.001 for leptin). In all categories of HOMA-IR, higher E-selectin and lower leptin concentrations were observed in men. Despite men’s higher Apo B values in each category of HOMA-IR compared with women, no trend across tertiles was verified. No statistical difference between the other biomarkers was found according to HOMA-IR tertiles (data not shown).

Correlations of interest regarding anthropometric measurements and plasma glucose with biomarkers were assessed for the total sample before variables were entered into the regression models. Correlations between waist circumference and Apo B (rho = 0.034, p = 0.490 and rho = 0.093, p = 0.037) and E-selectin (rho = 0.178, p < 0.001 and rho = 0.124, p = 0.004) were evaluated among men and women, respectively. BMI was directly correlated to leptin concentrations in men (rho = 0.363, p < 0.001) and women (rho = 0.425, p < 0.001). As expected, both adiposity parameters, BMI and waist circumference, were correlated to lipid and plasma glucose levels (data not shown). E-selectin was also correlated to plasma glucose in women (rho = 0.090, p = 0.038) but not men (rho = 0.059, p = 0.213).

Multiple regression analysis models were run separately for men and women using Apo B, leptin and E-selectin as dependent variables (Table 2) since these biomarkers differed between sexes in Table 1. All models included age, adiposity (waist circumference in apolipoprotein B and E-selectin models and BMI in the leptin model) and HOMA-IR. Among men, Apo B was associated with age independent of adiposity and the insulin resistance index. For both sexes, leptin was strongly associated with BMI and HOMA-IR independent of age. E-selectin was associated with waist circumference in the age-adjusted model but lost significance when HOMA-IR was included in the final model for both sexes.

**Table 2:** Linear regression models for apolipoprotein B, leptin or E-selectin (dependent variables) for men and women

Men												

	Apolipoprotein B				Leptin				E-selectin			
		
	r^2^	β	95% CI	p	r^2^	β	95% CI	p	r^2^	β	95% CI	p
Age	0.016	0.030	0.009–0.051	0.006	0.134	0.013	-0.004–0.029	0.127	0.008	0.004	-0.010–0.019	0.559
Adiposity*	0.018	-0.011	-0.023–0.001	0.065	0.184	0.068	0.044–0.092	0.000	0.011	0.005	-0.003–0.013	0.242
HOMA-IR	0.025	0.047	-0.023–0.117	0.189	0.150	0.067	0.013–0.120	0.015	0.046	0.076	0.028–0.124	0.002

Women												

	Apolipoprotein B				Leptin				E-selectin			
		
	r^2^	β	95% CI	p	r^2^	β	95% CI	p	r^2^	β	95% CI	p

Age	0.002	0.008	-0.012–0.028	0.430	0.156	-0.012	-0.026–0.004	0.142	0.008	-0.006	-0.021– 0.09	0.427
Adiposity*	0.002	0.001	-0.007–0.010	0.823	0.118	0.030	0.053–0.093	0.000	0.002	0.008	-0.005–0.010	0.542
HOMA-IR	0.020	-0.001	-0.086–0.085	0.988	0.165	0.093	0.028–0.158	0.005	0.028	0.097	0.033–0.160	0.003

CI: confidence interval.

All models included age and adiposity. * Waist circumference was used in apolipoprotein B and E-selectin models and BMI in the leptin model.

## DISCUSSION

Attempts to improve the prediction of cardiovascular events have motivated investigations into biomarkers involved in the early atherosclerotic process. Ageing is a major risk factor for atherosclerosis, and its manifestations in women are closely related to menopause. In the present study, a broad spectrum of circulating biomarkers of inflammation and endothelial dysfunction as well as traditional risk factors were examined in association with age and sex in a large sample of middle-aged adults of both sexes. As many of these markers track body adiposity, our study also calls attention to the influence of anthropometric measurements and the insulin resistance index. Our findings add new knowledge to these determinations: associations of Apo B with age and sex and of leptin and E-selectin with sex, adiposity and insulin resistance suggest that these demographic and metabolic characteristics should be taken into consideration when assessing these biomarkers for research or clinical purposes.

Atherogenesis starts early in life and accelerates with ageing. Our study reinforces that, in parallel, blood pressure levels, plasma glucose and lipids (except HDL-cholesterol) tend to increase in both sexes. In our female sample, the 45- to 54-year-old group showed higher BMIs and waist circumferences. The waist circumference value was higher in the oldest age group, confirming previous observations of central fat accumulating with age in men and women (31). Certainly, cytokines secreted by the adipose tissue contribute to disturbances of glucose and lipid metabolism and BP elevation with ageing (32). The deterioration of these traditional risk factors has common underlying mechanisms, such as inflammation and insulin resistance. Our data on increased IL-6 and TNF-α in older women and increased CRP in older men in our sample are in agreement with this pathophysiological pathway. Actually, inflammatory markers were shown to be correlated to Apo B, which is present in most atherogenic lipid particles in circulation. It is interesting that these results were found when the traditional risk factors were almost within the normal range (Table 1).

It was postulated that adhesion molecules, like selectins and CAMs, play a role in the development of the arterial plaque (33). We found that the greater concentrations of a soluble molecule that promotes leukocyte adhesion to endothelia in men is compatible with higher cardiovascular risk in this sex, particularly in the 35 to 54 age group. The association with sex was previously described in a sample of young individuals (21,22). Since the production of adhesion molecules is stimulated by metabolic disturbances (33), it was important to test the association of E-selectin with an insulin resistance index. Even after adjusting for adiposity and age, the correlation of E-selectin to HOMA-IR persisted. Other investigators had similar findings that showed that adhesion molecule concentrations were influenced by sex, age and HOMA-IR in individuals with low cardiometabolic risk (21,22). Apart from the limitations of the present study’s design, our findings could suggest that visceral adipose tissue might be influencing E-selectin synthesis, maybe through adipose tissue derived cytokines that induce insulin resistance. In animals, adipocyte was shown to express an adhesion molecule named ACAM (34). It was previously demonstrated that this endothelial, cell-specific adhesion glycoprotein is partially stimulated by inflammatory cytokines — like TNF-α and interleukins — produced by adipocytes, leukocytes and other cells (35). Transcription of the E-selectin gene is exclusively expressed on the endothelial cell surface and is mainly dependent on TNF-α-induced activation of the nuclear factor kappa B (35). For our knowledge, prospective studies have yet to prove whether circulating levels of E-selectin could be useful for identifying early abnormalities in the atherosclerotic process and whether different values should be considered for each sex.

Unadjusted analysis showed higher leptin concentrations in women, which was expected because it is an adipose tissue derived hormone. It is recognized that, at similar BMI levels, women have more fat mass than men (36). Leptin concentration is known to be associated with fat mass in women from puberty. Interestingly, higher mean values of leptin in the older group were only observed for men, which could be explained by their tendency to increase visceral adipose tissue with ageing. Interestingly, the association of leptin with body adiposity persisted after additional adjustment for insulin resistance index. This finding may indicate that such an association is highly dependent on the production of this hormone by the adipocyte and, to a lesser extent, related to the condition of insulin resistance.

On average, participants of the present study were overweight and 19% were obese. Being overweight is a recognized condition of insulin resistance (37) commonly accompanied by resistance to leptin (38). Insulin and leptin resistance are seen as the interface between inflammation and metabolism in obesity-related CVD. It would be reasonable to suppose that an increase in body adiposity with ageing might contribute to a deteriorating cardiovascular risk profile. However, this cross-sectional design precludes investigating a cause and effect relationship.

The findings of higher central adiposity, HOMA-IR and concentration of Apo B and E-selectin in men are compatible with the fact that the cardiovascular risk profile is worse in men than in pre-menopausal women (39,40). Our sample included a large number of middle-aged adults (younger than 55 years old), most of them women (55%), who did not have any overt CVD. Another advantage was the very low rate of hormone replacement therapy. Studies with these sample characteristics are relatively uncommon in the literature.

Our study has limitations. One major limitation is related to the high inter-individual, and possibly intra-individual, variability. Ideally, biomarkers should be determined on several occasions to obtain variability rates. Looking at the r^2^ values, concentrations of these biomarkers are weakly explained by the variables entered in the regression models. However, it is well known that age, sex and adiposity influence cardiovascular risk assessments. Our results suggest that these variables should also have an impact on some of the novel biomarkers. Therefore, they might be taken into account when their values are examined. The study design does not allow the establishment of a cause and effect relationship. Further investigation is needed to determine whether some novel cardiovascular biomarkers could contribute to earlier risk prediction needs.

In conclusion, our data indicate that age, sex, adiposity and, consequently, insulin resistance influence circulating levels of Apo B, leptin and E-selectin, thereby suggesting that those factors should be taken into consideration when assessing these parameters for research or clinical purposes in individuals with relatively low cardiometabolic risk.
